# Relying on Myself: The Lived Experience of Being at Risk for Falling in the Hospital Among Older Adults

**DOI:** 10.1093/geroni/igab046.3431

**Published:** 2021-12-17

**Authors:** Hanne Dolan, Cindy Rishel, Jessica Rainbow, Ruth Taylor-Piliae

**Affiliations:** University of Arizona, University of Arizona, Arizona, United States

## Abstract

Inpatient falls are a persistent problem and despite research efforts during the last decade, inpatient fall rates have not significantly decreased. Older adults have an estimated 50% greater inpatient fall rate than younger adults. How older adults perceive their own fall risk affects their adherence to fall prevention recommendations. The aim of this phenomenological study was to gain a deeper understanding of the lived experiences of being at risk for falling in the hospital among older adults aged 65 years and older (N=9). Participants (female=55%, age range=67 – 86) were interviewed twice using video conferencing within two weeks of hospital discharge. The audio-recorded interviews were transcribed, and then analyzed using van Manen’s interpretive phenomenological method. The Health Belief Model expanded with the concepts of independence, fear of falling, embarrassment, dignity, and positivity effect served as the theoretical framework. Five major interpretive themes emerged: Relying on Myself, Managing Balance Problems in an Unfamiliar Environment, Struggling to Maintain Identity, Following the Hospital Rules, and Maintaining Dignity in the Relationships with Nursing Staff. These themes describe how the participants thoughtfully planned their mobilization to avoid falls. This process was influenced by their struggling to remain independent, following the hospital fall prevention rules out of politeness, and experiencing both positive and negative relationships with nursing staff. Hospitalized older adults employed their self-efficacy to manage balance problems in the hospital. These findings have not been previously documented in the literature. Fall prevention interventions supporting hospitalized older adults’ self-management of fall risk are needed.

